# Multiplex Immunofluorescence: A Powerful Tool in Cancer Immunotherapy

**DOI:** 10.3390/ijms24043086

**Published:** 2023-02-04

**Authors:** Wenjie Sheng, Chaoyu Zhang, T. M. Mohiuddin, Marwah Al-Rawe, Felix Zeppernick, Franco H. Falcone, Ivo Meinhold-Heerlein, Ahmad Fawzi Hussain

**Affiliations:** 1 Department of Gynecology and Obstetrics, Medical Faculty, Justus-Liebig-University Giessen, Klinikstr. 33, 35392 Giessen, Germany; 2 Institute for Parasitology, Faculty of Veterinary Medicine, Justus Liebig University Giessen, 35392 Giessen, Germany

**Keywords:** multiplex, immunohistochemistry, fluorescence, antibody, cancer, immunotherapy

## Abstract

Traditional immunohistochemistry (IHC) has already become an essential method of diagnosis and therapy in cancer management. However, this antibody-based technique is limited to detecting a single marker per tissue section. Since immunotherapy has revolutionized the antineoplastic therapy, developing new immunohistochemistry strategies to detect multiple markers simultaneously to better understand tumor environment and predict or assess response to immunotherapy is necessary and urgent. Multiplex immunohistochemistry (mIHC)/multiplex immunofluorescence (mIF), such as multiplex chromogenic IHC and multiplex fluorescent immunohistochemistry (mfIHC), is a new and emerging technology to label multiple biomarkers in a single pathological section. The mfIHC shows a higher performance in cancer immunotherapy. This review summarizes the technologies, which are applied for mfIHC, and discusses how they are employed for immunotherapy research.

## 1. Introduction

Since Coons et al. described an immunofluorescence technique for detecting cellular antigens in mammalian tissue sections [1], immunohistochemistry (IHC) has become an essential diagnosis method in tissue pathology. This antibody-based technique has been further developed during the last decades on formalin-fixed, paraffin-embedded (FFPE) tissue specimens, which are ideal elements for studying solid tumors and hematologic malignancies. This technique is established as the gold standard tool for cancer diagnosis, provides valuable prognostic information, determines the appropriate therapy, and guides immunotherapy for a variety of tumor types [2,3,4,5].

However, identification of complex expression patterns for multiple biomarkers is often required in tissues such as sections of tumors to predict the response to and outcomes of immunotherapy. Traditional IHC has the main disadvantage that it only allows detection of a single biomarker per tissue section. Thus, developing multiple biomarkers detection within one section is necessary, especially for predicting the impact of immunotherapy.

Recently, multiplex immunohistochemistry/immunofluorescence (mIHC/IF) technologies are more and more discussed in immunotherapy because they enable simultaneous detection of multiple biomarkers in a single tissue section, including bright field and fluorescent field [6,7]. This comprehensive imaging gives a relative diagnostic accuracy of different biomarkers expression for clinical treatment [8], translational medicine [9], and precision medicine [10], and is even considered as the next-generation pathology [11]. This review summarizes the technologies of multiplexed fluorescent immunohistochemistry (mfIHC) as well as their application in research and clinical use, especially in cancer immunotherapy.

## 2. Fluorescent Immunohistochemistry

Fluorescent immunohistochemistry, or immunofluorescence staining (IF) is a special type of IHC, which utilizes a fluorescently labeled antibody to detect a target antigen. In general, there are two detection systems; the direct IF system, which uses fluorescence-labeled primary antibodies to bind target epitopes directly, and the indirect IF system, in which a secondary fluorescence-labeled antibody is employed to recognize the primary antibody bound to the target antigen. Since there is no need for secondary antibody incubation and washing steps, direct IF is less complicated, more specific, and timesaving. However, indirect IF has higher sensitivity since several secondary antibodies can bind to a single primary antibody, which amplifies the fluorescence signal. Furthermore, virtually all the same isotype primary antibodies from the same host species can be detected with an individual secondary antibody.

When more than one kind of fluorescent labeled antibody is applied, there are some technical issues such as spectral crosstalk among fluorescent dyes, cross-reactivity between antibodies, restrictive sample size, and fading of the fluorescent dyes as well as intrinsic autofluorescence of the tissue. This limits the number of antigens that can be detected simultaneously using fluorescent labeled antibodies [12]. Other limitations are the high cost and time consuming nature of this method. For clinical use, IF requires special equipment and training to allow pathologists to register histological diagnosis and identify specific tissue and cell type. Therefore, this method cannot support the robust generation of quantitative multiplexed data required to understand the relationship between tissue microarchitecture and expression at a single-cell level, which is particularly important for tumorigenesis, cancer development, and immunotherapy responses.

## 3. Multiplex Fluorescent Immunohistochemistry

**Figure 1:**
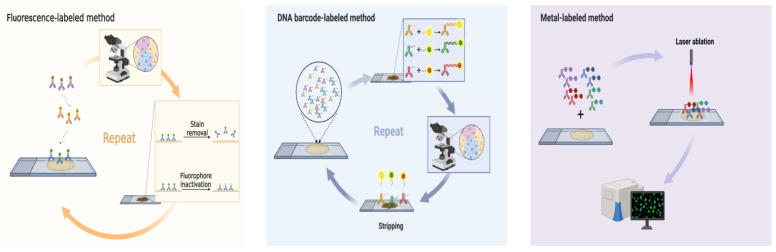
Schematic diagram showing the mechanism of each multiplex fluorescent immunohistochemistry method and was created with BioRender.com. Fluorescence-labeled method: multiepitope-ligand cartography (MELC), sequential immuno-peroxidase labelling and erasing (SIMPLE), iterative bleaching extends multiplexity (IBEX), multiplexed fluorescence microscopy (MxIF), cyclic immunofluorescence (CycIF), ChipCytometry, UltraPlex™, Opal™, quantum dots (QDs). DNA barcode-labeled method: DNA Exchange Imaging (DEI), codetection by indexing (CODEX), Immuno-SABER, Digital Spatial Profiling (DSP), InSituPlex^®^. Metal-labeled method: imaging Mass Cytometry (IMC), multiplexed ion beam imaging (MIBI). Created with BioRender.com.

Investigating multiple markers on a single tissue section and exhaustive study of cell component, cellular function and cell-cell interactions are necessary for accurate diagnosis and appropriate therapeutic strategies. Thus, a promising strategy called multiplex fluorescent immunohistochemistry (mfIHC) has been developed, which uses new multicolor immunohistochemistry methods, automated multispectral slide imaging, and advanced computer software (Figure 1). The mfIHC relies on direct or indirect detection of antigen as well, utilizing a fluorescence microscope to capture light emission with different spectral peaks against a dark background. Individual fluorophores are excited by one wavelength and emit at a longer specific wavelength (a phenomena known as Stokesshift) [13]. Subsequently, many mfIHC-related applications have been established for clinical research, especially immunotherapy [8,14,15].

Referring to mfIHC utilization for immunotherapy research, there are various methods depending on diverse principles. These can be classified into five classes: stain removal technologies, fluorophore inactivation technologies, multiplexed signal amplification, DNA barcoding technologies, and mass cytometry (Table 1).

### 3.1. Stain Removal Technologies

As one of the commonly used methods of mfIHC, stain removal technologies were developed with different platforms to study tumor tissue samples. The basic principle of these technologies is to clear the sample of one label, stain the sample with a new and different label, and repeat the process to detect multiple antigens in a single sample [16]. Here are different examples of stain removal technologies.

#### 3.1.1. Multiepitope-Ligand Cartography

Multiepitope-ligand cartography (MELC) or multi-epitope-ligand “Kartograph” (MELK) is an automated method to measure up to thousands of different classes, parts, or groups of molecules in liquid or solid samples, especially in cells and tissue specimens [17]. MELC can trace out the colocalization of several proteins in single sample of cells or tissue relying on sequential rounds of labeling biomarkers with fluorescent dyes [18]. In each cycle, the sample is incubated with fluorophore-labeled antibodies followed by acquisition of images by a high-sensitivity fluorescence microscope; finally, the sample is bleached at the excitation wavelength of the fluorescent dye, then a new staining cycle can be started [19].

With this approach, Schubert has analyzed the colocalization of 18 different cell surface proteins for cells and tissue sections in different compartments of the human immune system [20]. Further, this method was developed to detect up to 100 proteins within a single cell, which was called Toponome imaging systems (TIS) [21]. Moreover, MELC was also used for topological proteomics analysis to reveal the role of the adaptive immune system in colorectal cancer and select new antitumor immunotherapies [22].

However, the MELC method is constrained by the single microscope field of view instead of the whole section due to the photobleaching step, leading to the development of sequential immuno-peroxidase labelling and erasing (SIMPLE) [23].

#### 3.1.2. Sequential Immuno-Peroxidase Labelling and Erasing

Sequential immuno-peroxidase labelling and erasing (SIMPLE) was established by Glass et al. in 2009 [23]. This method is also based on stain removal technology, using the alcohol-soluble red peroxidase substrate 3-amino-9-ethylcarbazole (AEC) for at least parallel five markers visualization. The sample which is fixed and embedded is incubated with counterstaining primarily followed by a counterstain-only imaging. After antigen retrieval, immunohistochemical staining with AEC is performed, followed by full-slide scans. Next, AEC precipitate can be removed with 95% ethanol, and the antibodies can be removed in an elution solution as well. The sample is restained, and a multicolor composite image is generated.

**Figure 2:**
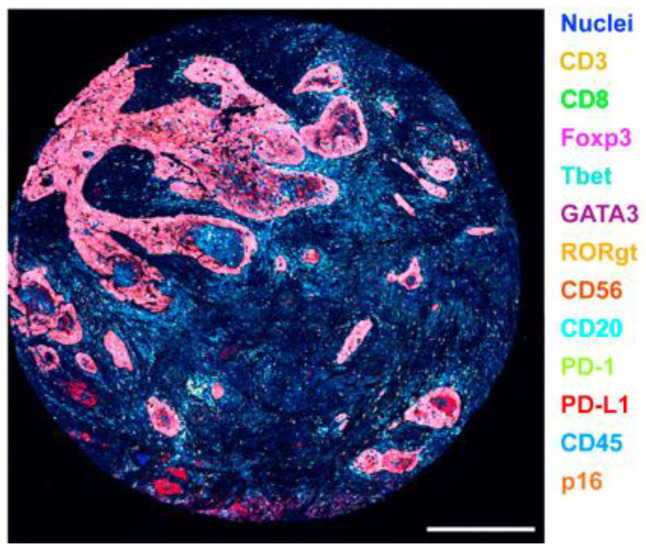
HNSCC FFPE sections stained with lymphoid biomarkers. Biomarkers and colors are shown on the right. Scale bar, 500 µm. (Reprinted with permission from [24]. Copyright 2017 Elsevier).

SIMPLE is often applied in immune profile in different cancer types. For example, using this method allowed to determine the correlation between immune complexity and the clinical outcomes as well as tumor subclassification in head and neck squamous cell carcinoma (HNSCC) (Figure 2). Additionally, it helped to indicate the therapeutic response to vaccination therapy in pancreatic ductal adenocarcinoma [24]. 

#### 3.1.3. Iterative Bleaching Extends Multiplexity

Iterative bleaching extends multiplexity (IBEX)is a recently emerging technology, which involves iterative immunolabeling and chemical bleaching method, enabling multiplexed imaging in various tissues [25]. In this method, specimens are prepared using tissue grossing protocols, then tissues can be prepared as FFPE tissues or as fixed frozen samples. The strong reducing agent lithium borohydride (LiBH_4_), which can bleach the fluorescence conjugated antibody plays an important role in IBEX [26]. Here, antibodies are conjugated with one of the following LiBH_4_-sensitive dyes: Pacific Blue, Alexa Fluor (AF)488, AF532, phycoerythrin (PE), AF555, eFluor (eF)570, iFluor (iF)594, AF647, eF660, AF680, AF700, or AF750, then exposed to LiBH_4,_ so that the fluorophore signal can be eliminated. On the other hand, antibodies conjugated with LiBH_4_-resistant dyes (normally Hoechst) maintain their signal over multiple bleaching and imaging cycles. IBEX can be performed either manually or automatically, and analyzed by SimpleITK open-source, without the need for programming skills [27].

IBEX enables high-resolution imaging of over 65 parameters simultaneously without physical damage. Therefore, it is a suitable method for revealing the complex cellular architecture and tumor immune interactions under a spatial context, contributing to tissue physiology and pathology. IBEX has been performed in mouse tissues (spleen, thymus, lung, small intestine, and liver) as well as human pancreatic lymph node with metastatic lesions sections [28]. In addition, Opal fluorophores and oligonucleotide-conjugated antibodies are also involved to further develop IBEX [28], and recently the 3D-IBEX method was applied in tissue sections [29]. Tables may have a footer.

### 3.2. Fluorophore Inactivation Technologies

The principle of this technology is roughly similar to stain removal technologies. However, it is does not rely on using different pH conditions, denaturation, or photobleaching to remove a stain, but on chemical inactivation to eliminate the fluorophore.

#### 3.2.1. Multiplexed Fluorescence Microscopy

Multiplexed fluorescence microscopy method (MxIF) is an imaging platform that enables 60 directly labeled antibodies to be applied to a single tissue section [30]. This method can provide quantitative, single-cell, and subcellular characterization of multiplexed molecular targets in FFPE tissue.

In MxIF, after acquiring background autofluorescence tissue images, the sample is stained with cyanine-based fluorescence-labeled antibody, then the images are captured followed by fluorescent dye inactivation using alkaline oxidation chemistry. This cycle can be repeated using different labeled antibodies. MxIF has analyzed the expression of 61 protein antigens in 747 colon cancer samples and even identified that placenta-specific 8 (PLAC8) as a molecule contributed to colon cancer invasion. Therefore, MxIF becomes a possible method for exploring the biological basis, drug distribution, and clinical diagnosis [31]. 

#### 3.2.2. Cyclic Immunofluorescence

Cyclic immunofluorescence (CycIF) follows the principle of MxIF. CycIF is a public-domain technology, which as the name suggests, involves repeated cycles of immunofluorescence staining and fluorophore inactivation [32]. Compared to MxIF, it allows to use general reagents and commercial antibodies without cyanine modification.

In general, there are three strategies to perform the CycIF. The most common one is staining the samples with up to three antibodies conjugated to different fluorescent dyes (Alexa Fluor dyes are recommended) with a counterstain. After inactivating the fluorophores by hydrogen peroxide and light as well as a wash step, another round of staining, imaging, and washing can be performed. CycIF has already been tested in adherent cells with up to five rounds and analyzed 15 antibody signals, and even 10 CycIF cycles can also be performed for strongly adherent cells [33].

**Figure 3:**
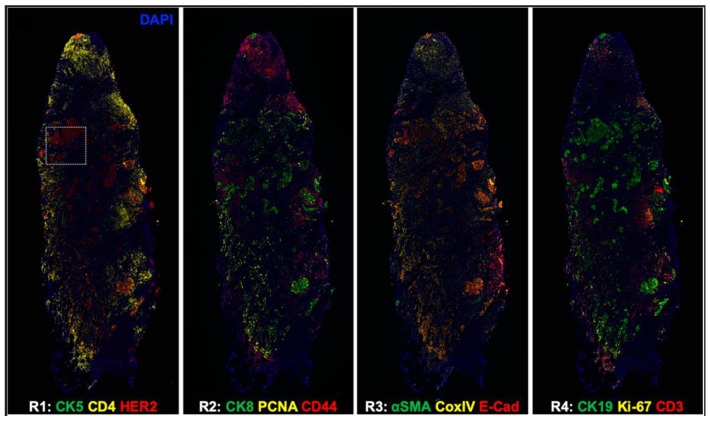
CycIF shows whole HER2 positive breast cancer tissue images. CK5, cytokeratin 5; CK8, cytokeratin 8; CK19, cytokeratin 19; PCNA, proliferating cell nuclear antigen; E-Cad, E-cadherin; α-SMA, α-smooth muscle antigen; CoxIV, cytochrome c oxidase. (Reprinted with permission from [36]. Copyright 2020 International Society for Optical Engineering).

For investigating responsiveness and resistance to therapy, a tissue-based cyclic immunofluorescence (t-CyCIF) method has been described in 2018. It showed that FFPE samples mounted on glass slides, which are the mostly used in histopathological diagnosis of cancer and other diseases, can create highly multiplexed immuno-fluorescence imaging by t-CyCIF. This technology allowed detection of over 60 different proteins in normal and tumor tissue samples from human patients, giving an efficient method for pre-clinical and clinical research [34]. Counting on the advantage of t-CyCIF that allowed to detect 60 antibodies and analyze FFPE in a single-cell level, the first evaluation of breast cancer-specific antibodies in a highly multiplexed imaging platform was performed. Epidermal growth factor receptor 2 (HER2), estrogen receptor (ER), and progesterone receptor (PR) have been validated to define the tumor microenvironment (TME) in breast cancers at a single-cell level [35]. To facilitate the demand of clinical histopathology, CycIF was combined with oligonucleotide conjugated antibodies, which were hybridized in situ with their complementary oligonucleotide sequence labeled with traditional fluorophores. This new strategy has generated up to 14 colors imaging of human breast cancer tissues (Figure 3) [36]. 

#### 3.2.3. ChipCytometry

As a notably advanced optical imaging-based platform for highly multiplexed tissue imaging (HMTI), ChipCytometry has extended the basic principle of t-CyCIF. FFPE sections undergo antigen retrieval before immobilizing them on the surface of the microfluidic chip, followed by recording the background autofluorescence. Subsequently, tissue sections are stained with up to five fluorophore-conjugated primary antibodies, then images are acquired. Fluorophores are finally bleached to prepare the tissue for the next staining cycle [37].

Using ChipCytometry, 30 different immune cells markers have been investigated. In addition, lineage-specific markers such as CD3, CD8, CD4, Foxp3, CD20, CD14/CD68, CD56, and the phenotypic markers CD45RA/CD45RO, PD-1, and Ki-67 were detected for exploring the cell heterogeneity [37]. Additionally, ChipCytometry was used to analyze PD-1 ligands (PD-L1 and PD-L2) on circulating tumor cells, which are associated with immune inhibitor therapy effectiveness [38].

### 3.3. Multiplexed Signal Amplification

To overcome the limitation of detecting low expressing antigens, multiplexed signal amplification has been developed. This technology includes multiplex modified hapten-based, tyramide signal amplification (TSA), and nanocrystal quantum dots. Here, the TSA is a commonly used one.

#### 3.3.1. Multiplex Modified Hapten-Based

Multiplex modified hapten-based (UltraPlex™) method can detect multiple biomarkers simultaneously by a standard two-step procedure. This method is independent of antibody species and generates 3–4 times stronger signals than direct fluorescence-tagged secondary antibodies, which are main advantages in mfIHC. In the UltraPlex™, tissue samples are incubated with the mix of primary antibodies for 1 h, and washed, then incubated with a panel of anti-hapten secondary antibodies for 1 h. After a washing step, slides are imaged by fluorescence microscopy or digital slide scanning [39]. UltraPlex™ has developed three primary antibodies (anti-CD8, anti-PD-L1, and anti-panCK) in a single NSCLC tissue to define the TME [40].

#### 3.3.2. Tyramide Signal Amplification (TSA)

**Figure 4:**
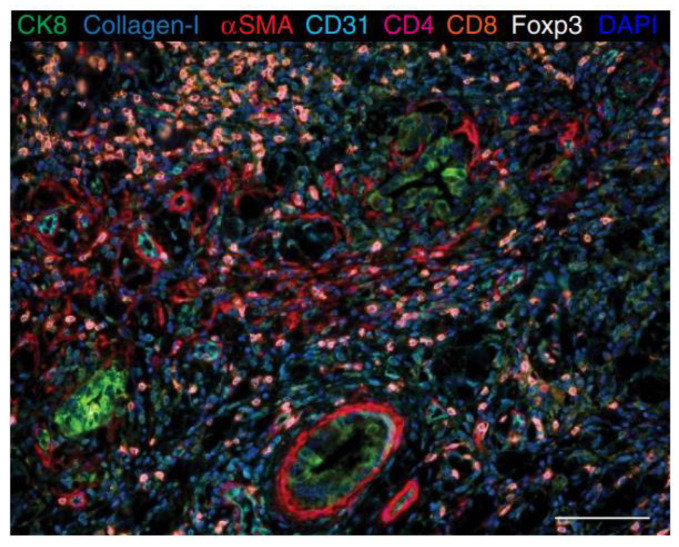
Opal™ shows multiplex staining in human pancreatic ductal adenocarcinoma (PDAC) tissue sections. CK8 (green), Collagen-I (extracellular teal blue), aSMA (cytoplasmic, red), CD31 (membrane, cyan), CD4 (membrane, pseudocolored magenta), CD8 (membrane, Cy5, orange), Foxp3 (nuclear, white), autofluorescence (black), and the DAPI nuclear marker (blue). Scale bar 100 µM. (Reprinted with permission from [46], Copyright 2017 Springer Nature under a Creative Commons Attribution 4.0 International License).

TSA is an enzyme-mediated method that catalyzes the deposition of tyramide from low to large amount in an immunoassay system [41]. Tyramide can be biotinylated or labeled with fluorescent dye and catalyzed by streptavidin–horseradish peroxidase enzyme (HRP) [13]. Subsequently, multiple tyramide-labeled molecules are laid down at the site of the epitope. TSA can provide a systematic evaluation of different processes in different tumor tissues. Parra et al. have used this technology to stain about 4000 FFPE tumor samples for translational research and explained the cancer biology at a protein level and identified therapeutic targets and biomarkers [42]. TSA can be used to stain up to eight markers on a single slide (Figure 4). Researchers could design the panel, which required selecting and detecting for their projects (e.g., immune cell populations, T-cell behaviors, and myeloid cell populations). After image analysis, individual marker expression and cell phenotypes were identified, then helped to characterize immune cells and relevant checkpoint proteins [43].

Relying on this signal amplification technology, Perkin Elmer developed the Opal™ workflow, which utilizes individual TSA-conjugated fluorophores to detect various targets within mfIHC assays. This method allows multiple primary antibodies from individual species to be detected without species cross-reactivity, based on an antibody stripping protocol (microwave treatment), which removes primary antibodies and secondary HRP-conjugated antibodies. Opal™ assay has been used to stain immune cell populations for various markers (e.g., CD3, CD4, CD8, CD20, CD25, CD68, CD69, FOXP3, PD-1, Tim-3 and Ki-67, or any other combination) on a single tissue slide with a multiplex antibody panel to evaluate complex phenotypes in the TME [13] and supported the evaluation of PD-L1 expression in metastatic gastric cancer treatment [44].

However, staining tissues with this assay generates a high level of complexity and requires intensive work to optimize each step in order to limit epitope damage and signal loss during the sequential staining protocol. Moreover, equal HRP concentration contributes to prevent TSA dimer formation, and titration of primary and secondary reduced dimer formation as well. Therefore, some improvements for the Opal method have been explored [15,45].

#### 3.3.3. Nanocrystal Quantum Dots

This technology relies on quantum dots (QDs), which are fluorescent nanoparticles (2–10 nm in diameter) [47] that represent a special property such as a thin, adjustable, and symmetric emission fluorescence spectrum and is photochemically stable compared with traditional fluorophores [48]. Moreover, different QDs fluorescence can be excited by a single light source in the meantime, with minimum overlap between spectra and no endogenous autofluorescence from tissue sections [49], which offers a distinct advantage for multiplexed targets detection. In this technology, secondary antibodies are labeled with QDs to detect targets. After targeted samples are exposed under a light source, the fluorescence is captured and analyzed.

Within QDs technology, Peng et al. showed the co-evolution of cancer cells, as well as their microenvironment on tissues in order to understand the complex process of cancer invasion [50]. Even though HER2 was expressed at a lower level in some breast cancer cells, it could be also detected clearly and showed multiplexed QDs-based imaging [51].

### 3.4. DNA Barcoding Technologies

This set of technologies takes full advantage of DNA characteristics and extends the capability of mfIHC.

#### 3.4.1. DNA Exchange Imaging

DNA Exchange Imaging (DEI) is a generalization of developed Exchange-PAINT technology, which is a DNA-based multiplex method enabling fluorescence-labeled ligands to bind to target molecules in super resolution [52]. In DEI assay, antibodies are conjugated with short DNA oligos (usually 9–10 nucleotides) called docking strands, followed by image acquisition, where fluorescence-labeled complementary imager strands are applied sequentially, and signals are removed by rapid buffer exchange after each cycle of imaging. DEI allows labeling multiplex protein targets by single-step simultaneous immunostaining [53].

Compared to other methods, which need long multi-rounds of immunostaining, DEI assay can be carried out within a short time. Moreover, DEI can be applied to a wide range of microscopy platforms such as standard resolution exchange-confocal and various super-resolution methods. Although there are few cancer-related research publications using this method, DEI is a promising technique to study the drug discovery and clinical pathology for immunotherapy.

#### 3.4.2. Codetection by Indexing

Codetection by indexing (CODEX) is another DNA barcoding technique for highly multiplexed cytometric imaging. Unlike the other methods, the antibodies are labeled with DNA oligonucleotides, instead of fluorophores or rare metal elements. Samples are stained with DNA oligonucleotide-labeled antibodies simultaneously, then fluorescence-labeled oligonucleotides, which are complementary with those conjugated oligonucleotides, are added. Three fluorescence-labeled tissues can be imaged by conventional fluorescence microscopy subsequently in one cycle. After washing away the fluorophores, samples can be stained in the next cycle for a different nucleotide. Finally, each group of antibodies in each cycle is visualized at a known, pre-defined cycle of the indexing protocol and the multiparameter image is reconstructed [54].

**Figure 5:**
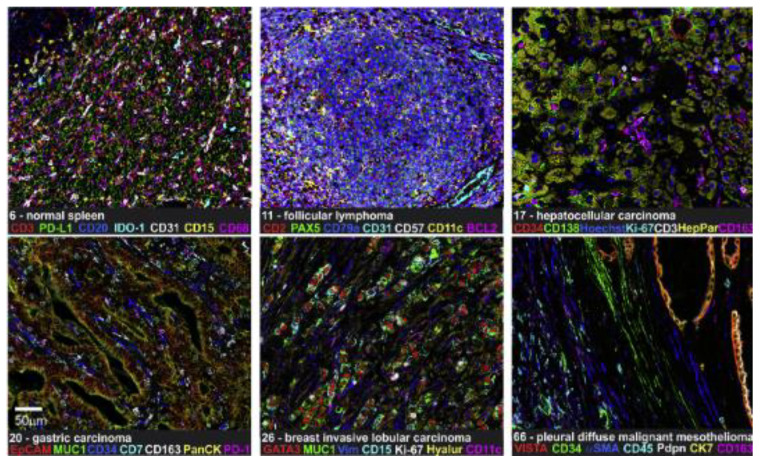
CODEX shows 7-plex imaging in different FFPE tissues. IDO-1, indoleamine 2,3-dioxygenase 1; Vim, vimentin; VISTA, V domain immunoglobulin suppressor of T-cell activation. Scale bar 50 µm. (Reprinted with permission from [55]. Copyright © 2020, Elsevier under Creative Commons CC-BY license).

CODEX is already commercialized and owned by Akoya Biosciences [54]. Schürch and his colleagues re-designed CODEX to be suitable for FFPE tissue and tissue microarrays (Figure 5). This alternative method allowed to profile 140 tissue regions from 35 advanced-stage colorectal cancer patients with 56 protein markers simultaneously, and better understood an interaction between antitumor immunity and the immune tumor microenvironment (iTME) [55]. These researchers also have optimized the CODEX protocol, including conjugate purified antibody to DNA oligonucleotides, validation conjugated antibodies by CODEX staining, and performing CODEX multi-cyclic imaging in FFPE and fresh frozen tissues [56]. Moreover, a study included eight immunoregulatory proteins (ICOS, IDO-1, LAG-3, PD-1, PD-L1, OX40, Tim-3, and VISTA) to simultaneously phenotype, localize, and quantify these functional molecules on individual cells within the TME by CODEX, providing important insights for cancer immunotherapy [57].

#### 3.4.3. Signal Amplification by Exchange Reaction

One limitation of DEI and CODEX is that DNA strands, which are conjugated to primary antibodies directly, lack secondary antibodies for signal amplification, especially in low abundance target detection for tissues. To overcome this limitation, the signal amplification by exchange reaction (SABER) method was developed [58]. Based on SABER technology, immunostaining with signal amplification by exchange reaction (Immuno-SABER) can achieve highly multiplexed signal amplification in situ without enzymatic reactions. Firstly, samples are stained with multiple DNA-barcoded primary antibodies, then these barcodes are hybridized with orthogonal single-stranded DNA concatemers by primer exchange reaction (PER). Finally, fluorophore-labelled DNA imager strands hybridize to the repeated binding sites on the concatemers, generating an amplified signal. To realize the unlimited targets detection at once, a process called Exchange-SABER, which can image sequentially by hybridization and dehybridization of orthogonal imagers in multiple rapid exchange cycles [58].

Various samples, including cultured cells, cryosections, FFPE sections, and whole mount tissues have been validated with the signal amplification from 5- to 180-fold with Immuno-SABER. SABER was also applied in oligo-based FISH (Fluorescence in situ hybridization) probes [59], combined with quantum dot (QD-SABER) [60]. Importantly, SABER has been cited by the NIH Common Fund Human Biomolecular Atlas Program as a transformative technology, which will be used to construct a three-dimensional molecular and cellular atlas of the human body [61].

#### 3.4.4. Digital Spatial Profiling (DSP)

Digital Spatial Profiling (DSP) technology is based on a UV-photocleavable oligonucleotide tag conjugated to an antibody or mRNA hybridization probes, used to stain samples and focused UV light releases oligonucleotides from any region of interest (ROI). These oligonucleotides can be collected and subsequently counted using the Nanostring Barcode system (GeoMx^TM^) and image up to four channels; after extensive washing, UV exposure and oligonucleotides collection are repeated [62].

For DSP application in clinical research, Blank et al. have explained the combination immunotherapy superiority for melanoma treatment [63], and later multiple biomarkers (CD3, CD4, CD8, CD20, and PD-L1)**,** which could be potentially available for predicting response to immune therapy in non–small cell lung cancer (NSCLC), were evaluated [64]. Because DSP has the property of single-cell sensitivity, it was combined with next-generation sequencing (NGS) to analyze 1412 genes (4998 RNA probes). Meanwhile, the spatial identification of 44 proteins and 96 genes (928 RNA probes) in lymphoid, colorectal tumor and autoimmune FFBE samples has been revealed by the nCounter system [62]. The DSP technology has the potential for greater multiplexing using NGS readout, and to be a tool widely used in both research and medical care.

#### 3.4.5. InSituPlex^®^

InSituPlex^®^ is a method also based on antibodies conjugated to unique DNA barcodes (oligonucleotides). After FFPE section dewaxing and antigen retrieval (heat-induced epitope retrieval) using the traditional IHC protocol, samples are incubated with mixing primary antibodies conjugated to barcodes. Next, all labeled antibodies are processed to amplify DNA barcodes simultaneously. Finally, the mixture of complementary oligonucleotide probes tagged with fluorophores are incubated with those processed samples for hybridizing and labeling the targets, then acquiring fluorescent imaging [65].

InSituPlex^®^ overcomes the low signal amplification, long-lasting workflow, and tissue-damaging risks, so it is a very promising technology in immuno-oncology research. Using this method, Singhal et al. showed the subcellular distribution of the multi-functional transcriptional regulator, Kaiso (*ZBTB33*), and its correlation with immune-suppressive characteristics and defined Kaiso’s value in breast cancer progression, which has potential as a predictive biomarker to guide future treatment, especially the immunotherapy using immune checkpoint inhibitors [66].

### 3.5. Mass Cytometry

For decades, flow cytometry has been used as a crucial method to investigate the cellular networks on single-cell level. However, the limited number of fluorophores to be detected simultaneously in flow cytometry has become a disadvantage of multiparameter assessment for cellular processes. Therefore, another format for flow cytometry called mass cytometry was developed, meanwhile the Cytometry by Time of Flight (CyTOF) was introduced [67]. Unlike traditional flow cytometry, which utilizes the fluorophores as reporters, antibodies are labeled with metal isotopes in mass cytometry. Samples are stained with these antibodies, and after nebulizing, ionizing, and atomizing, isotopes can be identified and quantified by CyTOF [68]. Based on mass cytometry technology, several methods have been developed such as imaging mass cytometry (IMC) and Multiplexed Ion Beam Imaging (MIBI).

#### 3.5.1. Imaging Mass Cytometry

**Figure 6:**
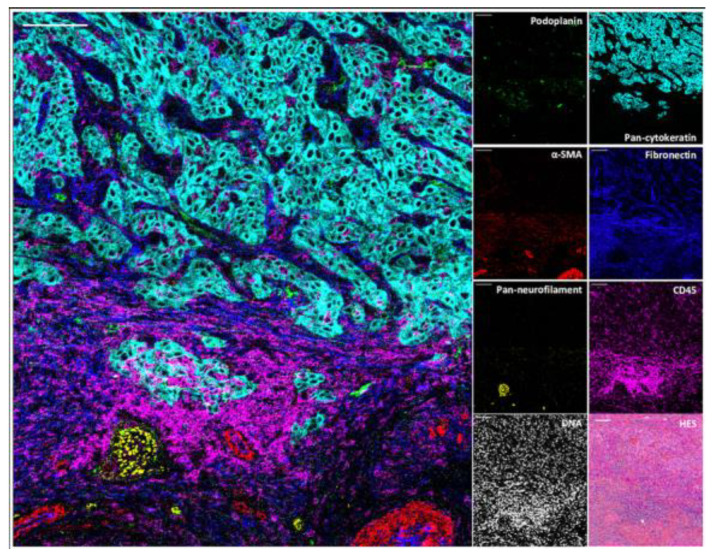
IMC shows the structural and cellular iTME constitutions in the cutaneous squamous cell carcinomas (cSCC), representing lymphatic vessels (podoplanin), blood vessels and cancer-associated fibroblasts (αSMA), nerve fibers (pan-neurofilament), tumor cells (pan-cytokeratin), extracellular matrix (fibronectin) and immune cells (CD45), nuclei, and hematoxylin-eosin-saffron (HES) also were done in the same region. Scale bar 100 µm. (Reprinted with permission from [75]. Copyright 2021 Frontiers Media SA).

Imaging mass cytometry (IMC) is an optimized mass cytometry, which is applied in immunocytochemistry and immunohistochemistry. Owning to a high-resolution laser ablation system, IMC can reveal the spatial information, allowing the description of cell subtypes and cell–cell interactions and emphasizing tumor heterogeneity (Figure 6). After labeling with metal isotypes, samples are ablated with a laser beam and transferred to the CyTOF spot by spot, and finally the epitope expression determined by CyTOF [69].

The application of IMC for the individual cell’s analysis has been extended after Gerdtsson et al. published their study [70]. For example, IMC was performed the complex breast cancer single-cell phenotypes and their spatial context, showing the multicellular features of the TME and subtypes of breast cancer, as well as the association with clinical outcomes and patient-specific possibilities [71]. This technology also allowed over 40 protein markers simultaneously on tissue sections with subcellular resolution [69]. Sandara et al. have evaluated 25 targets simultaneously on pretreatment FFPE tissue samples from immunotherapy-treated melanoma patients [72]. To define immune cell interactions in the TME, and access the response to immunotherapy, IMC investigated immune cell markers and chemokine ligands from melanoma samples [73] and was firstly applied in a novel case report of sarcomatoid urothelial carcinoma (SUC) for investigating the immune cell repertoire and PD-L1 expression [74].

#### 3.5.2. Multiplexed Ion Beam Imaging

Multiplexed Ion Beam Imaging (MIBI) is very similar to IMC but utilizes secondary ion mass spectrometry to image antibodies tagged with isotopically pure elemental metal reporters. Samples are incubated with primary antibodies conjugated to stable lanthanides that are highly enriched for an individual isotope. These prepared samples, which have rasterized oxygen duoplasmatron primary ion beam characters, provide free lanthanide adducts of the bound antibodies as secondary ions. In the end, those metal-conjugated antibodies are quantified by secondary ion beam imaging (SIMS).

Since MIBI can analyze up to 100 targets simultaneously over a five-log dynamic range, Michael et al. studied FFPE human breast tumor tissue sections stained with 10 labels at the same time, providing new insights into disease pathogenesis [14]. Subsequently, Keren et al. developed multiplexed ion beam imaging by time of flight (MIBI-TOF), an analysis instrument instead of SIMS that up to 36 labeled antibodies simultaneously, exhibiting regional variability in tumor cell phenotypes for immune response and revealing the complex tumor immune landscape [76,77]. In 2020, MIBI-TOF was combined with the single-cell metabolic regulome profiling (scMEP) method, which based on CyTOF to reveal the spatial texture of cellular metabolism in human tissue with 36 antibodies directly, providing the possibility of analyzing metabolic conditions from existing clinical cohorts, then predicting the cancer outcomes and therapeutic effect [78].

To analyze cell function more precisely for clinical applications, Xiavier et al. presented high-definition multiplex ion beam imaging (HD-MIBI) technology. It contributed to visualization of the relationship between multiple biomolecules and their ligands or small molecules down to ~30 nm lateral resolution. With this technology, the process of biomolecule and drug distributions can be imaged in biologically relevant subcellular microenvironments, which allows a greater understanding of chemotherapeutic treatment resistance [79].

## 4. Conclusions

In contrast to traditional fluorescent immunohistochemistry, mfIHC has a significant advantage, as it allows multiple targets to be detected simultaneously. Compared with standard cancer treatments such as chemotherapy, radiotherapy, and surgery, cancer immunotherapy has realized a longer survival time and a better quality of life and radically changed the treatment and prognosis of many cancers. However, a significant proportion of patients treated with cancer immunotherapy do not benefit or derive a limited benefit from this therapy. This is mainly due to choosing the wrong immune checkpoint inhibitors, their combinations, and/or the administration time point. In this context, predicting the tumor response for immunotherapies individually or in combination and immune-related adverse events associated with these treatments are required to avoid overtreatment of immune checkpoint inhibitors and minimize its adverse events.

As the next-generation technology, more and more mfIHC methods are expected to be developed for preclinical research and clinical application have increased extraordinarily in the last five years, to reach a better understanding of tumorigenesis, tumor development, and response to immunotherapy either pre- or post-treatment. This research also included PD-1/PD-L1 immune checkpoint inhibition, which is considered a breakthrough in cancer immunotherapy, but still some patients do not respond. mfIHC technologies have contributed to the personalized treatment, which translational medicine and precision medicine are working on (Table 2).

**Figure 7:**
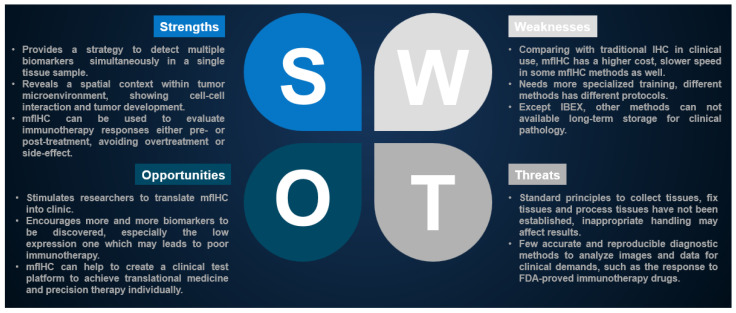
Strengths, Weaknesses, Opportunities, and Threats (SWOT) analysis regarding mfIHC applications in clinical cancer immunotherapy.

However, development of a new method needs multiskilled collaboration, including pathologists, oncologists, immunologists, and molecular biologists, and even more, well training with imaging analysis software. Moreover, the stained samples should be storable for a long time to cater for clinical demands and follow-up studies (Table 3). Thus, there is an urgent need to develop simple, fast, efficient, automated, and inexpensive investigative tools for immune cell profiling, which allows predicting the tumor response for immunotherapy reagents and their synergistic effects as well as associated immune-related adverse events (Figure 7).

## Figures and Tables

**Table 1:** Overview the mfIHC technologies.

Method Name	Vendor Name	Sample	Maximal Labeling	Direct/ Indirect Detection	Antibody Conjugation	Stain Removal Method	Time Consuming (Per Cycle) *	Resolution
Stain removal technologies								
MELC (Toponome imaging systems)	ToposNomos GmbH	FFPE	100	Direct	Fluorescent based	Bleaching	two proteins per hour (one tag/one dye per cycle)	<40 nm
SIMPLE	NA	FFPE	12	Indirect	Fluorescent based	Alcohol-soluble red peroxidase substrate AEC method	3 h	15–20 μm
IBEX	NA	Frozen/FFPE	>65	Direct/indirect	Fluorescent based	LiBH4-based bleaching	Manual ~3.5 h/automated ~1.5 h	160 nm
Fluorophore inactivation technologies								
MxIF	Cell IDx	FFPE	60	Indirect	Fluorescent based	Alkaline oxidation chemistry inactivation	1 h 15 min	1 μm
CycIF	NA	FFPE	60	Direct	Fluorescent based	Hydrogen peroxide and light inactivation	~24 h	5 μm
Chip Cytometry	Zellsafe™	Cell suspensions /frozen/FFPE	60	Direct	Fluorescent based	Chemical bleaching or light photobleaching	~1 h	5 μm
Multiplexed signal amplification								
Multiplex modified hapten-based	UltraPlex™	FFPE	4	Indirect	Fluorescent based	Antibody stripping	2 h	NA
TSA	Roche and Akoya Biosciences	Cell suspensions /FFPE	9	Indirect	Fluorescent based	Antibody stripping	1 h	0.25–0.9 μm
QDs	NA	FFPE	5	Direct/Indirect	Fluorescent based	Chemical bleaching	6 h	Super resolution
DNA barcoding technologies								
DEI	NA	FFPE	8	Indirect	DNA-barcoding based	NA	2–3 h	20 nm
CODEX	Akoya Biosciences	Cell suspensions/frozen/FFPE	60	Indirect	DNA-barcoding based	NA	<1 day (Whole slide imaging)	260 nm
Immuno-SABER	NA	Cell suspensions/frozen/FFPE	10	Indirect	DNA-barcoding based	NA	1 h	Super resolution
DSP	NanoString	Frozen/FFPE	96	Indirect	DNA-barcoding based	NA	1–2 h	10 μm
InSituPlex^®^	Ultivue	FFPE	15	Indirect	DNA-barcoding based	NA	5.5 h	NA
Mass cytometry								
IMC	Hyperion	Cell suspensions /frozen/FFPE	>40	Direct	Metal-based	NA	2 weeks (0.5 mm × 0.5 mm ROI takes ~3.5 h with a slide scanner)	1 μm
MIBI	Ionpath	Cell suspensions /FFPE	40–100	Direct	Metal-based	NA	2 weeks (Whole slide imaging)	260 nm

* Due to different section size, image acquisition time is various, this is a rough estimate.

**Table 2:** Applications of mfIHC technologies clinical studies.

Method Name	Cancer Type	Biomarkers Studies	Refs.
Stain removal technologies			
MELC (Toponome imaging systems)	Colorectal cancer	CD3, CD4, CD25, CD29, CD44, human lymphocyte antigen (HLA)-DR	[22]
SIMPLE	HNSCC PDAC	CD3, CD4, CD8, CD46, CD68, PD-1, Ki67, Eomes-odermin, GrzB, IDO, Tbet	[24]
IBEX	NA	NA	NA
Fluorophore inactivation technologies			
MxIF	Colon cancer	ER, androgen receptor (AR), p53, Her2, PLAC8	[30,31]
CycIF	Breast cancer	Her2, ER, PR	[35]
ChipCytometry	Breast cancer	PD-L1, PD-L2	[38]
Multiplexed signal amplification			
Multiplex modified hapten-based	NSCLC	CD8, PD-L1, and panCK	[40]
TSA	Metastatic gastric cancer (GC)	PD-L1	[44]
QDs	Gastric cancer/ breast cancer	type IV collagen, macrophages, matrix metalloproteinase 9 (MMP9), CD105	[50]
	Breast cancer	type IV collagen, Her2	[51]
DNA barcoding technologies			
DEI	N	NA	NA
CODEX	Cutaneous T cell lymphoma (CTCL)	ICOS, IDO-1, LAG-3, PD-1, PD-L1, OX40, Tim-3, VISTA	[57]
Immuno-SABER	NA	NA	NA
DSP	NSCLC	CD3, CD4, CD8, CD20, PD-L1	[64]
InSituPlex^®^	Breast cancer	Kaiso	[66]
Mass cytometry			
IMC	Melanoma	MHC-I, HMB45, S100, IFNGR1, IRF1, CD45RO, PD-L1, CD163, B7-H3, LAG3, TIM3, FOXP3, CD4, B7-H4, CD68, PD-1, CD20, CD8, PD-1H, Ki67, B2M, CD3a, CSF1R, PD-L2, Granzyme B, MHC-II, CXCL9, CXCL10, CXCL13	[72,73]
	SUC	PD-1, PD-L1	[74]
MIBI	Breast cancer	double-stranded DNA (dsDNA), ERα, PR, E-cadherin, Ki-67, vimentin, actin, keratin, HER2, PD-1, PD-L1	[14,77]

**Table 3:** Advantages and disadvantages for different mfIHC technologies.

Method Name	Advantage	Disadvantage
Stain removal technologies		
MELC (Toponome imaging systems)	Detects hundreds of proteins and high resolution	The multiprobe image is limited to a single microscopic medium-to-high power field and high cost
SIMPLE	Easy to perform by whole-slide scanner and can be labeled primary antibodies from same species	Up to 12 biomarkers
IBEX	Allows over 65 biomarkers to detect and compatible with over 250 commercial antibodies	Not commercialized and few studies
Fluorophore inactivation technologies		
MxIF	Up to 60 biomarkers	Time-consuming and relatively expensive
CycIF	Use commonly reagents and instruments	Before the next staining, coverslip should be removed and time-consuming
ChipCytometry	Detects unlimited number of biomarkers, long-storage samples, removes autofluorescence and instrument automaticity	Damage the tissue adherence and photobleachable dyes may generate weak signals during imaging processing
Multiplexed signal amplification		
Multiplex modified hapten-based	Two-hour fast staining and cocktail antibodies are used in a single slide	Maximal four biomarkers can be labeled per slide and not applied widely
TSA	Avoids antibody cross-reactivity and may realize an automated protocol	Nine biomarkers can be labeled per slide
QDs	Removes autofluorescence and has much stronger signals	Big size relatively, has toxicity and limited nanocrystals
DNA barcoding technologies		
DEI	Short-time staining and applies for most microscopy platforms	Lack of signal amplification, few studies
CODEX	Allows 60 biomarkers labeled and can be imaged by conventional fluorescence microscopy, also keeps the morphology of normal and diseased tissues	Longer scanning and lack of signal amplification
Immuno-SABER	High multiplexing, sensitivity and 5–180-fold signal amplification	Up to 10-plex and few publications
DSP	No-damage staining protocol and performs high multiplexing image on FFPE samples	Chooses ROI manually and is not able to reconstruct images
InSituPlex^®^	Good signal in low-expression antigen, 5.5 h workflow and relatively cheap	Few studies
Mass cytometry		
IMC	Removes autofluorescence, reveals the quantity of proteins in subcellular level	Lack of signal amplification, the rate of image acquisition is slow and relatively low resolution in subcellular level
MIBI	A large number of metal-antibodies can be labeled spectral overlap and high resolution	Time-consuming, instrument and metal-antibodies are expensive

## Data Availability

Not applicable.
